# Acute effects of using an electronic nicotine-delivery device (electronic cigarette) on myocardial function: comparison with the effects of regular cigarettes

**DOI:** 10.1186/1471-2261-14-78

**Published:** 2014-06-23

**Authors:** Konstantinos E Farsalinos, Dimitris Tsiapras, Stamatis Kyrzopoulos, Maria Savvopoulou, Vassilis Voudris

**Affiliations:** 1 Department of Cardiology, Onassis Cardiac Surgery Center, Sygrou 356, Kallithea 17674, Greece

**Keywords:** Electronic cigarette, Smoking, Myocardial function, Diastolic function, Tobacco harm reduction, Nicotine

## Abstract

**Background:**

Electronic cigarettes have been developed and marketed in recent years as smoking substitutes. However, no studies have evaluated their effects on the cardiovascular system. The purpose of this study was to examine the immediate effects of electronic cigarette use on left ventricular (LV) function, compared to the well-documented acute adverse effects of smoking.

**Methods:**

Echocardiographic examinations were performed in 36 healthy heavy smokers (SM, age 36 ± 5 years) before and after smoking 1 cigarette and in 40 electronic cigarette users (ECIG, age 35 ± 5 years) before and after using the device with “medium-strength” nicotine concentration (11 mg/ml) for 7 minutes. Mitral flow diastolic velocities (E, A), their ratio (E/A), deceleration time (DT), isovolumetric relaxation time (IVRT) and corrected-to-heart rate IVRT (IVRTc) were measured. Mitral annulus systolic (Sm), and diastolic (Em, Am) velocities were estimated. Myocardial performance index was calculated from Doppler flow (MPI) and tissue Doppler (MPIt). Longitudinal deformation measurements of global strain (GS), systolic (SRs) and diastolic (SRe, SRa) strain rate were also performed.

**Results:**

Baseline measurements were similar in both groups. In SM, IVRT and IVRTc were prolonged, Em and SRe were decreased, and both MPI and MPIt were elevated after smoking. In ECIG, no differences were observed after device use. Comparing after-use measurements, ECIG had higher Em (P = 0.032) and SRe (P = 0.022), and lower IVRTc (P = 0.011), MPI (P = 0.001) and MPIt (P = 0.019). The observed differences were significant even after adjusting for changes in heart rate and blood pressure.

**Conclusions:**

Although acute smoking causes a delay in myocardial relaxation, electronic cigarette use has no immediate effects. Electronic cigarettes’ role in tobacco harm reduction should be studied intensively in order to determine whether switching to electronic cigarette use may have long-term beneficial effects on smokers’ health.

**Trial registration:**

Current Controlled Trials ISRCTN16974547

## Background

Smoking is a major risk factor for cardiovascular disease [1,2]. Although several pharmaceutical products are available for smoking cessation, long term quit-rates are relatively low [3]. Therefore, tobacco harm reduction strategy and products have been developed, with the main goal to reduce the amount of harmful substances administered to the human body.

Electronic cigarettes have been introduced to the market in recent years as an alternative-to-smoking habit. They consist of a battery-part, a cartridge containing liquid and an electrical resistance that is heated by activation of the battery and evaporates the liquid. The liquid usually contains glycerol, propylene glycol, water, nicotine and a variety of flavours that the user can choose. By using this device, nicotine is delivered to the upper and lower respiratory tract without any combustion involved. Millions of people are using electronic cigarettes worldwide; however, lack of clinical research has raised global debate, controversy and serious public health concerns [4].

Several studies have shown that, even in healthy smokers, acute smoking inhalation has significant adverse effects on left ventricular (LV) myocardial function that can be detected by echocardiography [5-7]. No study has ever evaluated the effects of electronic cigarette use on cardiac function; thus, the purpose of the current study was to investigate the acute effects of using an electronic cigarette ad lib for 7 minutes on haemodynamic parameters and myocardial function, compared to the effects of smoking a tobacco cigarette.

## Methods

### Study sample

The study sample consisted of consecutive healthy subjects visiting our hospital for routine examinations that volunteered to participate. All participants were asymptomatic, had normal physical examination and resting electrocardiogram and were not taking any medications. Smokers (group SM) were included if they were smoking for at least 5 years and were consuming at least 15 cigarettes per day. The reason for including only heavy smokers was that a study examining the characteristics of electronic cigarette consumers showed that most electronic cigarette users were formerly heavy smokers [8]. Electronic cigarette users (group ECIG) were included if they had quit smoking and were using electronic cigarettes with nicotine-containing liquid for at least 1 month, according to self-report. To avoid potential compensatory effects from using lower nicotine-containing liquid, participants were included if they were daily consumers of similar “strength” liquids (9-12 mg/ml nicotine concentration) to that used in the study (11 mg/ml). Exclusion criteria were: presence of any major risk factor for cardiovascular disease (i.e. diabetes, hypertension, hyperlipidemia and family history of premature coronary artery disease), history of endocrine disorders, body-mass index > 30 kg/m2 and more than occasional alcohol intake. Additional exclusion criteria were derived from the echocardiography studies: elevated LV mass index (>115 g/m2 for males and > 95 g/m2 for females), abnormal LV function (LV ejection fraction < 55%) and more than mild valve regurgitation.

In total, 81 subjects were eligible to participate. Three smokers did not present for the scheduled evaluation. One electronic cigarette user was excluded because of moderate aortic regurgitation and ascending aorta dilatation due to bicuspid aortic valve. One smoker was excluded due to mildly depressed ejection fraction and hypokinesia of LV lateral wall. The final study sample consisted of 76 subjects, 40 electronic cigarette users (3 females) and 36 smokers (3 females). Written informed consent was obtained from all subjects for participation in the study, and the protocol was approved by the ethics committee of Onassis Cardiac Surgery Center.

## Materials

**Figure 1:**
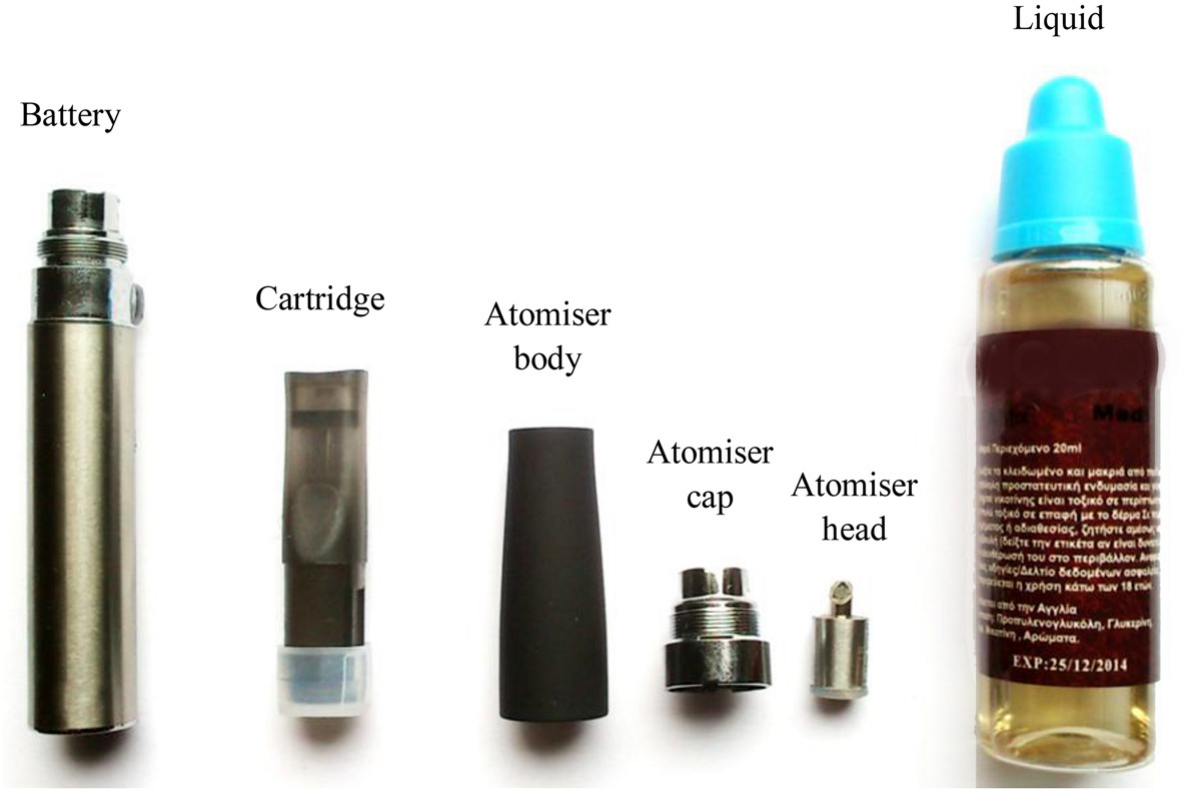
Electronic cigarette device and liquid used in the study.

All smokers were asked to use one commercially-available tobacco cigarette of the same nicotine (1.0 mg), tar (10 mg) and carbon monoxide (10 mg) yields. Electronic cigarette users were asked to use a commercially-available device with liquid containing 11 mg/ml nicotine concentration. The device used was an eGo-T battery (Nobacco, Athens, Greece) with an eGo-C atomiser (Alter Ego, Athens, Greece). It is considered a “second-generation” device. Unlike cigarette-like devices which consist of a small battery and a polyfil-containing atomiser (commonly called “cartomiser”), the electronic cigarette used in this study is a multi-piece system (Figure 1). It consists of a 650 mAh rechargeable lithium battery, delivering 3.5 volts to the atomiser (measured by a volt-meter), and an atomiser consisting of 4 parts: the tank which stores the liquid (capacity of approximately 1.1 ml), the atomiser body, the atomiser head which includes the resistance, and the atomiser cap. It is a manually-activated device, by pressing a button; it does not produce any vapour when not activated by the user.

The electronic cigarette liquid used in the study contained 11 mg/ml nicotine and is considered “medium strength” according to manufacturer’s report (USA Mix Med, formerly known as MLB-Med, Nobacco, Athens, Greece). It is sold in 20 ml bottles. It was the only liquid tested by an independent laboratory (National Center for Scientific Research “Demokritos”, mass spectrometry and dioxin analysis laboratory) at the time of study initiation [9]. According to the laboratory report, the contents were: propylene glycol (α -propylene glycol or 1,2-propanediol) in a concentration > 60%, linalool (3,7-dimethylocta-1,6-dien-3-ol) in a concentration < 5%, nicotine (<10%), tobacco essence (<5%), and methyl vanillin (4-hydroxy-3-methoxybenzaldehyde) at < 1%. No tobacco-specific nitrosamines or polycyclic aromatic hydrocarbons were detected.

For every participant, a new cartridge and atomiser head was used. One of the researchers filled the cartridge with 1 ml of liquid; subsequently it was positioned in the atomiser and the participant started using it. The battery was fully charged before being used by each subject.

### Study protocol

Participants presented to the echocardiographic laboratory after fasting and refraining from alcohol and caffeine consumption for 4 hours; they were also asked to refrain from smoking and electronic cigarette use for 4 hours before the study.

Participants were allowed to rest for 5 minutes before initiating the echocardiographic examination. A baseline echocardiographic examination was performed in smokers, who were then transferred to a room next to the echocardiography laboratory and smoked 1 tobacco cigarette. For electronic cigarette users, after the baseline echocardiogram they were asked to use the electronic cigarette device ad lib for 7 minutes in another room which was not used by smokers, to avoid environmental exposure to smoke. Subsequently, all participants returned to the echocardiography laboratory and, after 5 minutes of rest, a second echocardiogram was performed in both groups.

Heart rate and BP were measured before and during each echocardiographic examination. The Brinkman index was calculated (product of number of cigarettes smoked daily and years of smoking) according to participants’ self-report. Echocardiograms were performed using a commercially available system (Vivid 7, GE Vingmed, Horten, Norway). Studies were digitally recorded on hard disk for offline analysis using dedicated software (Echopac, GE Medical Systems, Horten, Norway) by a single, blinded to the protocol, experienced echocardiographer. Reported values represent the average of 3 consecutive beats.

### Two-dimensional echocardiographic measurements

The echocardiographic examinations were performed according to recent guidelines [9]. LV dimensions, septal and posterior wall thickness were measured from standard 2-dimensional images at parasternal long-axis view. LV mass was indexed to body-surface area. Ejection fraction was evaluated from the apical four and two-chamber views using the Simpson’s rule [10]. Left atrial (LA) antero-posterior diameter was also measured.

### Doppler flow and tissue Doppler velocity measurements

From transmitral flow measurements, peak early (E) and late (A) velocities, their ratio (E/A) and E wave deceleration time (DT) were estimated. Ejection time was estimated by recording LV outflow tract velocity. By simultaneously recording aortic and mitral flows using continuous-wave Doppler the isovolumetric relaxation time (IVRT) was measured, and was then corrected to heart rate by dividing it with the square root of R-R interval (IVRTc).

Pulsed-wave Doppler tissue velocities were measured by placing a 1.5 mm sample volume at the lateral, septal, anterior and inferior insertion sites of the mitral leaflets. Systolic (Sm), early diastolic (Em) and late diastolic (Am) peak velocities were measured and averaged from the 4 sites. The ratio of early-to-late annular velocity (Em/Am) and early mitral flow to early diastolic mitral annular velocity (E/Em) were also determined.

**Figure 2:**
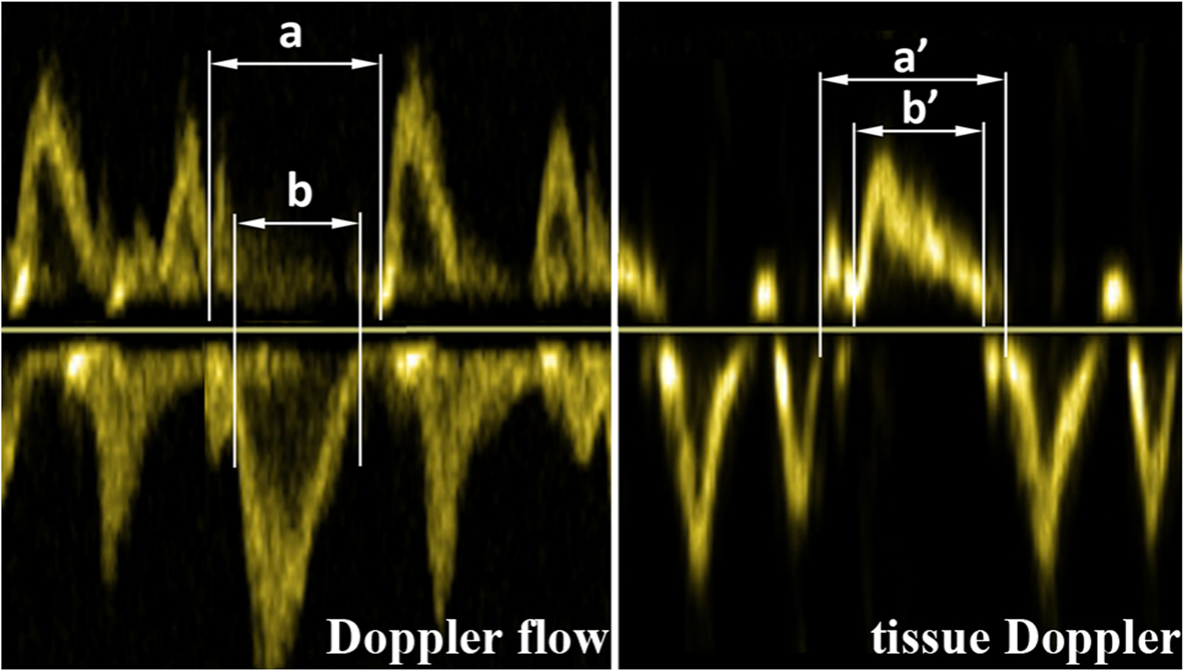
Myocardial performance index, measured by two methods: (1) Doppler flow velocity measurements of mitral inflow and left ventricular outflow tract; the index was derived by the formula: MPI = (a-b)/b, and (2) Pulsed-wave tissue Doppler measurements of mitral annulus velocity; the index was derived from the formula: MPIt = (a’-b’)/b’.

Myocardial performance index was measured by two methods (Figure 2): using Doppler flow velocity measurements as described by Tei et al. [11] (MPI) and using pulsed-wave tissue Doppler measurements of mitral annulus velocities (MPIt) [12].

To check for reproducibility of measurements, the intraobserver mean percent error (the absolute difference between two measurements divided by their mean) was calculated from 10 randomly selected studies 15 days later, analyzed by the same blinded echocardiographer who performed all measurements. The results were 5.1 ± 2.9% for IVRT, 3.5 ± 2.5% for MPI, 3.6 ± 2.2% for MPIt and 2.6 ± 1.9% for Em.

### Longitudinal deformation measurements

Longitudinal deformation measurements were performed by analyzing two-dimensional echocardiographic images using the method of speckle tracking echocardiography [13]. End-diastole was defined as the peak of the R wave on the electrocardiographic trace; end-systole (aortic valve closure) was defined from pulsed-wave Doppler tracing at the LV outflow tract as the end of systolic forward flow. Subjects with inadequate tracking of more than one LV segment in each view were excluded from the analysis. By averaging segmental values in all views, end-systolic global strain (GS) was measured. Global peak longitudinal systolic (SRs), early diastolic (SRe) and late diastolic (SRa) strain rate were measured. The intraobserver mean percent error of longitudinal deformation measurements in our laboratory was 3.1 ± 1.5% for GS, 3.6 ± 1.8% for SRs, 3.9 ± 1.9% for SRe and 3.6 ± 2.0% for SRa.

### Statistical analysis

The Kolmogorov-Smirnov tests were applied to assess the normality of data; all parameters were normally distributed except from daily cigarette consumption. Continuous variables were expressed as mean ± SD or median (interquartile range). Categorical variables were expressed as number (percentage). Inter-group comparisons of baseline characteristics data were made by unpaired Student’s t-test and Mann–Whitney test; Fisher’s exact test was used for categorical variables.

Repeated measurements analysis of variance (ANOVA) was used in order to evaluate changes in parameters before and after smoking one cigarette or using the electronic cigarette device (before-use and after-use measurements). Changes in echocardiographic and deformation parameters that were significantly different between the two study groups from analysis of variance were further analyzed using linear regression analyses, in order to find if the effect of smoking was significant after adjusting for changes in heart rate and systolic BP. For every parameter, a different linear regression analysis was performed. Change (Δ) in parameter was the dependent variable; group (SM vs. ECIG) and change in heart rate and systolic BP were the independent variables. All P values reported are two-tailed. Statistical significance was set at 0.05 and analyses were conducted using SPSS statistical software (version 18.0, SPSS Inc., Chicago, USA).

A repeated measures ANOVA power analysis was conducted. For this design, 76 participants (40 in the smokers group and 36 in the electronic cigarette users group) achieved a power of 0.90 for the between-subjects main effect at an effect size of 0.30; a power of 0.90 for the within-subjects main effect at an effect size of 0.15; and a power of 0.90 for the interaction effect at an effect size of 0.15.

## Results

Both groups had similar baseline characteristics (Table 1). Electronic cigarette users had quit smoking for 97 ± 50 days and were using electronic cigarettes for 100 ± 49 days. They had higher lifetime smoking exposure, with Brinkman index 33% higher compared to smokers, due to higher daily cigarette consumption when they were smokers.

**Table 1:** Baseline characteristics of the study population

**Characteristic**	**Smokers (n = 36)**	**Electronic cigarette users (n = 40)**	**P-value**
Males n (%)	32 (88.9)	36 (90)	1.000^a^
Age (years)	36 ± 5	35 ± 5	0.764
Body mass index (kg/m^2^)	24.8 ± 2.3	25.3 ± 2.4	0.304
Body surface area (m^2^)	2.03 ± 0.15	2.00 ± 0.18	0.322
Smoking duration (years)	16 ± 5	17 ± 5	0.571
Cigarette consumption (n/d)^b^	20 (20–26)	30 (20–35)	0.004^c^
Brinkman index	371 ± 132	493 ± 228	0.005
Electronic cigarette use duration^d^		6 ± 4	
Systolic BP (mmHg)	123.0 ± 9.8	123.9 ± 8.6	0.653
Diastolic BP (mmHg)	75.8 ± 5.6	75.6 ± 6.1	0.834
Heart rate (beats/m)	67.5 ± 7.9	67.1 ± 10.3	0.841
Pressure-rate product	8308 ± 1235	8312 ± 1363	0.989
Glucose (mmol/l)	4.51 ± 0.34	4.44 ± 0.35	0.410
Total cholesterol (mmol/l)	4.85 ± 0.21	4.77 ± 0.30	0.177
LDL cholesterol (mmol/l)	2.99 ± 0.23	2.91 ± 0.26	0.175
HDL cholesterol (mmol/l)	1.38 ± 0.15	1.38 ± 0.18	0.943
Triglycerides (mmol/l)	1.05 ± 0.14	1.04 ± 0.18	0.693
Ejection fraction (%)	63 ± 5	62 ± 4	0.463
LA diameter (mm)	35 ± 4	34 ± 3	0.359
LV mass index (g/m^2^)	64 ± 10	65 ± 13	0.663

BP, blood pressure, LVEDV, left ventricular end-diastolic volume; LVESV, left ventricular end-systolic volume; LDL, low-density lipoprotein; HDL, high-density lipoprotein; LA, left atrium.

^a^Fisher’s exact test; ^b^Values expressed as median (interquartile range); ^c^Mann-Whitney test; ^d^Duration expressed in months.

Changes in haemodynamic, Doppler echocardiography and longitudinal deformation measurements for the study groups are presented in Tables 2 and 3. Baseline measurements were similar between groups for all parameters.

**Table 2:** Haemodynamic and Doppler flow measurements in electronic cigarette users (ECIG, n = 40) and smokers (SM, n = 36), before and after device and cigarette use respectively

**Parameter**	**Before use**	**After use**	**Change**	**P-value**^**a**^	**P-value**^**b**^
**Systolic BP (mmHg)**					
ECIG	123.9 ± 8.6	124.6 ± 9.9	0.7 ± 4.6	0.374	< 0.001
SM	123.0 ± 9.8	129.6 ± 9.2	6.6 ± 5.2	< 0.001	
P-value^c^	0.653	0.025			
**Diastolic BP (mmHg)**					
ECIG	75.6 ± 6.1	78.5 ± 5.9	3.0 ± 3.6	< 0.001	0.079
SM	75.8 ± 5.6	80.2 ± 5.8	4.4 ± 3.3	< 0.001	
P-value^c^	0.834	0.209			
**Heart rate (beats/m)**					
ECIG	67.1 ± 10.3	67.5 ± 10.6	0.4 ± 4.8	0.649	< 0.001
SM	67.5 ± 7.9	73.5 ± 6.8	5.9 ± 4.7	< 0.001	
P-value^c^	0.841	0.005			
**Pressure-rate product**					
ECIG	8312 ± 1363	8397 ± 1462	84 ± 708	0.456	< 0.001
SM	8308 ± 1235	9556 ± 1084	1248 ± 840	< 0.001	
P-value^c^	0.989	< 0.001			
**E (cm/s)**					
ECIG	70.1 ± 12.5	71.4 ± 13.2	1.2 ± 5.0	0.130	0.132
SM	72.9 ± 8.5	72.2 ± 10.2	-0.6 ± 6.1	0.565	
P-value^c^	0.268	0.756			
**A (cm/s)**					
ECIG	51.1 ± 10.2	52.7 ± 9.8	1.6 ± 5.6	0.083	0.317
SM	50.4 ± 8.8	53.3 ± 9.1	2.9 ± 5.7	0.007	
P-value^c^	0.774	0.764			
**E/A**					
ECIG	1.41 ± 0.29	1.37 ± 0.26	-0.03 ± 0.14	0.171	0.053
SM	1.49 ± 0.32	1.39 ± 0.30	-0.10 ± 0.16	0.001	
P-value^c^	0.235	0.809			
**DT (ms)**					
ECIG	173 ± 11	174 ± 14	1 ± 8	0.581	0.570
SM	170 ± 16	172 ± 16	3 ± 10	0.086	
P-value^c^	0.448	0.719			
**IVRT (ms)**					
ECIG	74.6 ± 9.5	73.6 ± 9.9	-1.0 ± 5.7	0.275	0.001
SM	73.0 ± 8.7	77.7 ± 13.5	5.6 ± 9.2	< 0.001	
P-value^c^	0.450	0.132			
**IVRTc (ms)**					
ECIG	78.9 ± 11.8	77.7 ± 11.6	-1.2 ± 6.9	0.286	< 0.001
SM	77.3 ± 10.1	86.1 ± 16.4	10.4 ± 10.1	< 0.001	
P-value^c^	0.524	0.011			
**MPI**					
ECIG	0.39 ± 0.07	0.38 ± 0.06	-0.01 ± 0.04	0.330	0.001
SM	0.40 ± 0.05	0.43 ± 0.06	0.03 ± 0.04	0.002	
P-value^c^	0.355	0.001			

BP, blood pressure; E, mitral flow early diastolic velocity; A, mitral flow late diastolic velocity; DT, deceleration time of early mitral flow; IVRT, isovolumetric relaxation time; IVRTc, IVRT corrected to heart rate; MPI, myocardial performance index estimated by Doppler flow echocardiography.

^a^P-value for time effect.

^b^Repeated measurements ANOVA. Effects reported are significant differences between the two groups in the degree of change in each particular variable.

^c^P-value for group effect.

**Table 3:** Tissue Doppler velocity and longitudinal deformation measurements in electronic cigarette users (ECIG, n = 40) and smokers (SM, n = 36), before and after device and cigarette use respectively*

**Parameter**	**Before use**	**After use**	**Change**	**P-value**^**a**^	**P-value**^**b**^
**Sm (cm/s)**					
ECIG	9.7 ± 1.6	9.9 ± 1.6	0.2 ± 0.7	0.171	0.613
SM	9.7 ± 1.4	9.7 ± 1.5	-0.8 ± 1.1	0.571	
P-value^c^	0.896	0.723			
**Em (cm/s)**					
ECIG	12.7 ± 1.9	12.9 ± 2.1	0.2 ± 0.7	0.095	< 0.001
SM	12.8 ± 2.1	11.9 ± 1.5	-0.7 ± 1.4	< 0.001	
P-value^c^	0.892	0.032			
**Am (cm/s)**					
ECIG	9.7 ± 1.7	9.9 ± 1.6	0.2 ± 0.8	0.122	0.441
SM	9.3 ± 1.2	9.4 ± 1.3	0.1 ± 0.6	0.801	
P-value^c^	0.212	0.099			
**Em/Am**					
ECIG	1.34 ± 0.29	1.33 ± 0.28	-0.01 ± 0.13	0.540	0.011
SM	1.40 ± 0.28	1.30 ± 0.24	-0.08 ± 0.13	0.004	
P-value^c^	0.408	0.655			
**E/Em**					
ECIG	5.60 ± 1.04	5.61 ± 1.11	0.01 ± 0.47	0.869	0.052
SM	5.83 ± 0.95	6.10 ± 0.98	0.29 ± 0.74	0.021	
P-value^c^	0.311	0.044			
**MPIt**					
ECIG	0.48 ± 0.08	0.47 ± 0.09	-0.01 ± 0.04	0.080	< 0.001
SM	0.49 ± 0.06	0.52 ± 0.07	0.03 ± 0.05	0.004	
P-value^c^	0.654	0.019			
**GS (%)**					
ECIG	-21.1 ± 1.9	-21.5 ± 1.6	-0.4 ± 1.2	0.059	0.087
SM	-21.0 ± 2.6	-20.7 ± 3.1	0.2 ± 1.7	0.441	
P-value^c^	0.769	0.192			
**SRs (s**^**-1**^**)**					
ECIG	-1.13 ± 0.10	-1.14 ± 0.11	-0.01 ± 0.07	0.362	0.613
SM	-1.08 ± 0.13	-1.10 ± 0.13	-0.2 ± 0.1	0.150	
P-value^c^	0.059	0.115			
**SRe (s**^**-1**^**)**					
ECIG	1.47 ± 0.25	1.49 ± 0.23	0.01 ± 0.08	0.347	< 0.001
SM	1.43 ± 0.25	1.35 ± 0.24	-0.08 ± 0.12	< 0.001	
P-value^c^	0.493	0.022			
**SRa (s**^**-1**^**)**					
ECIG	0.88 ± 0.20	0.89 ± 0.18	0.01 ± 0.08	0.462	0.441
SM	0.86 ± 0.14	0.88 ± 0.14	0.03 ± 0.09	0.111	
P-value^c^	0.536	0.796			

*Longitudinal deformation measurements were performed in 37 electronic cigarette users and 34 smokers.

Sm, mitral annulus systolic velocity; Em, mitral annulus early diastolic velocity; Am, mitral annulus late diastolic velocity; MPIt, myocardial performance index estimated by tissue Doppler echocardiography; GS, global longitudinal strain; SRs, peak systolic strain rate; SRe, peak early diastolic strain rate; SRa, peak late diastolic strain rate.

^a^P-value for time effect.

^b^Repeated measurements ANOVA. Effects reported are significant differences between the two groups in the degree of change in each particular variable.

^c^P-value for group effect.

After-use values of systolic BP, heart rate and pressure-rate product were elevated in the SM group but not in the ECIG group (Table 2). The overall change from baseline was significantly different between the two groups. In contrast, diastolic BP increased equally in both groups.

From Doppler flow echocardiographic measurements (Table 2), E velocity and DT remained unchanged after use in both groups. A velocity was increased and E/A was decreased in SM, but the overall change was not significantly different between the two groups (P = 0.317 and P = 0.053, respectively). IVRT, IVRTc and MPI were increased after smoking one cigarette in the SM group, and the degree of change was significantly different between the two study groups (P = 0.001, P < 0.001 and P = 0.001 respectively). The after-use levels of IVRTc and MPI were greater in SM compared to ECIG, as was shown by the between-groups analysis.

Concerning Doppler tissue velocity measurements (Table 3), Sm and Am remained unchanged after use in both groups. However, Em was significantly reduced in SM group after smoking. It was lower when compared to ECIG after using the device, and the degree of change was significantly different between the two groups (P < 0.001). Em/Am was reduced and E/Em was increased in SM, but the difference of the overall change between the two groups was statistically significant for Em/Am only (P = 0.011). MPIt increased after smoking in SM; the degree of change was significantly different between the two groups (P < 0.001), with after-use levels being significantly higher in SM compared to ECIG (P = 0.019).

Longitudinal deformation measurements (Table 3) were feasible in 37 electronic cigarette users and 34 smokers. No difference in GS, SRs and SRa was observed in ECIG and SM after use. However, SRe was significantly reduced in SM post-smoking, with the degree of change being statistically significant between groups (P < 0.001).

The results of multiple linear regression analyses are displayed in Table 4. Even after adjusting for changes in systolic BP and heart rate, changes in IVRT, IVRTc, MPI, Em, MPIt and SRe were significantly higher in SM group.

**Table 4:** **Results from linear regression analyses for the effect of group (smokers vs. electronic cigarette users) on changes (**Δ**) of Doppler echocardiography measurements, after adjusting for changes in systolic blood pressure and heart rate**

**Dependent variable**	β*	SE**	**P-value**
ΔIVRT (ms)	4.64	2.12	0.032
ΔIVRTc (ms)	5.46	2.34	0.022
ΔMPI	0.03	0.01	0.013
ΔEm (cm/s)	-0.87	0.25	0.001
ΔMPIt	0.04	0.01	0.001
ΔSRe (s^-1^)	-0.06	0.03	0.039

*Regression coefficient for the comparison of SM group to ECIG group, adjusted for changes in systolic blood pressure and heart rate.

**Standard Error.

## Discussion

This is the first study to examine the acute effects of electronic cigarette use on myocardial function. No adverse effects on LV myocardial function were observed after using electronic cigarette with nicotine-containing liquid for 7 minutes. On the contrary, significant changes in diastolic function parameters were found after smoking 1 tobacco cigarette.

The acute adverse effects of smoking on myocardial relaxation were originally observed in coronary artery disease patients [14], with acute impairment of coronary vasomotion implicated as the main cause [15]. Such effects on diastolic function are also detected in healthy smokers [5-7] Cigarette smoke contains significant amounts of free radicals, promoting oxidative stress and inflammation [16] At the cellular level, decreased function of myocardial mitochondria [17] and DNA damage [18] has been observed. These mechanisms may be implicated in delaying myocardial relaxation from acute use and promoting atherosclerosis and cardiovascular disease from chronic use. In this study, several parameters commonly used for evaluating diastolic function [19] and longitudinal deformation measurements which are considered more sensitive in detecting pathology [20] were significantly altered after smoking inhalation.

Electronic cigarettes were invented in 2003, but awareness and use has significantly increased over the past 3 years [21]. They do not contain tobacco and their use does not involve combustion. However, lack of research on their health effects has generated significant controversy over their safety. FDA and WHO issued public statements in 2009, expressing concern and recommending that electronic cigarette use should be avoided. WHO has specifically asked for studies to be performed before regulation or even ban is imposed. Cahn and Siegel summarized the results of 16 studies evaluating the chemical composition of liquids used for electronic cigarettes [22]. Nitrosamines were found in only two of the studies, at levels similar to those present in nicotine patch; a recent review indicated that the levels of nitrosamines in electronic cigarettes were up to 1800 times lower compared to tobacco cigarettes [23]. The main constituents, besides nicotine, were propylene glycol and glycerine, which are also present in tobacco cigarettes; however, the combustion process from smoking leads to production of acrolein, acetaldehyde and formaldehyde, which promote oxidative stress and have cardiotoxic properties [24]. In electronic cigarettes, such chemicals may be formed from the heating process during liquid evaporation; however, the levels found were lower compared to tobacco cigarettes by orders of magnitude [25]. This may explain the results from laboratory studies, in which electronic cigarette vapour was significantly less cytotoxic compared to cigarette smoke on cultured cells [26,27]. Cardiotoxic substances like nitrosamines, heavy metals and polycyclic aromatic hydrocarbons were not detected in the liquid used in this study [9]. These parameters may explain the differences in diastolic function observed between smokers and electronic cigarette users after smoking and device use. Moreover, a study evaluating the effects of smoking compared to nicotine delivered by gum showed that nicotine alone did not cause acute changes in diastolic function [28]. It seems that nicotine absorption rate is lower from electronic compared to tobacco cigarette use [29], even when using new-generation devices [30]; the difference in haemodynamic response between the two groups may be attributed to this. However, haemodynamic parameters cannot explain the differences in diastolic function parameters, since linear regression analyses revealed that changes in Doppler and deformation parameters were associated with cigarette smoking even after adjusting for changes in systolic BP and heart rate.

From a public health perspective, epidemiological studies have shown that tobacco harm reduction strategy and products may be promising regarding cardiovascular disease risk reduction [31]. Electronic cigarettes are unique since they are the only products that do not contain tobacco, while they mimic the act of smoking and provide motor and sensory stimulation. Thus, they may deal with both the chemical (nicotine delivery) and behavioural components of cigarette addiction [22] and studies indicate that they may be effective in promoting smoking cessation [32,33]. This study provides the first clinical evidence that electronic cigarettes have less acute adverse effects on myocardial function when compared to tobacco cigarettes.

Some limitations apply to this study. A small sample size was studied, and examination focused only on immediate effects. The results do not indicate that electronic cigarettes are absolutely safe for the cardiovascular system. Other parameters known to be adversely affected by acute smoking, such as coronary microvascular and endothelial function or vascular distensibility, were not examined. Moreover, the parameters examined are affected mainly by heart rate changes. Although heart rate was not included as a covariate in the repeated-measures ANOVA, the linear regression analysis showed that changes in diastolic function were significantly different between groups independently of the changes in heart rate and systolic BP. This can be explained by the small difference in post-use heart rate between groups of only 6 beats per minute. Studies on long-term effects are necessary; however, more time of use is needed before any such studies are published since electronic cigarettes were introduced to the market in recent years and there is a substantial delay between smoking initiation and development of clinically-evident disease. We asked subjects to use the electronic cigarette for 7 minutes. It is unknown whether more time of use could have had a different impact. However, timing was based on the approximate time of smoking 1 regular cigarette; in fact, it took smokers 5 minutes to smoke one cigarette while electronic cigarette users were asked to use the device for a longer time. Additionally, experienced users were examined, who use the device more intensively than novice users [34]. Unfortunately, there are no other means of comparing electronic with tobacco cigarette use. Although plasma nicotine levels were not measured, the haemodynamic response observed suggests that the nicotine delivery rate from electronic cigarettes is lower and slower compared to tobacco cigarettes. This has been validated by studies performed recently [30,35]. The results of this study are not necessarily applicable to all liquids available in the market. If non-pharmaceutical grade nicotine is used, several tobacco impurities may be present and inhaled by the user. The same applies for other liquid constituents [21]. Finally, although all subjects were considered healthy based on history taking, clinical examination, resting ECG and echocardiogram, it cannot be excluded that some subjects may suffer from subclinical coronary artery disease. However, there was no indication to perform any additional examinations in the study population.

## Conclusions

Although acute smoking inhalation caused a delay in LV myocardial relaxation in smokers, electronic cigarette use was found to have no such immediate effects in daily users of the device. This short-term beneficial profile of electronic cigarette compared to smoking, although not conclusive about its overall health-effects as a tobacco harm reduction product, provides the first evidence about the cardiovascular effects of this device. Since awareness and use of electronic cigarettes are continuously rising, more studies are urgently needed, focusing on the pathophysiological mechanisms of disease where smoking is implicated and ultimately on long-term effects. Such studies will provide additional scientific data to public health authorities so that they decide on the regulatory status of this product.

## Competing interests

After this study was completed, the authors have performed studies using funds provided to the institution by e-cigarette companies.

## Authors’ contributions

KF was responsible for study conception and design. KF, DT and MS were responsible for data collection. SK was responsible for off-line measurements of echocardiographic parameters. KF, DT and VV were responsible for statistical analysis and interpretation. KF, DT and VV drafted the manuscript. All authors read and approved the manuscript.

## Pre-publication history

The pre-publication history for this paper can be accessed here:

http://www.biomedcentral.com/1471-2261/14/78/prepub
